# Clinical efficacy and acceptability of remote fetal heart rate self-monitoring in Southern China

**DOI:** 10.1186/s12884-023-05985-9

**Published:** 2023-10-07

**Authors:** Yujie Gan, Caixia Zhu, Yueqin Zhou, Jieying Wu, Fenge Cai, Qiang Wu, Jingwan Huang, Yanna Zhu, Haitian Chen

**Affiliations:** 1 Department of Obstetrics and Gynecology, Zhongshan Boai Hospital, Zhongshan, China; 2 Department of Obstetrics and Gynecology, The First Affiliated Hospital of Sun Yat-Sen University, Guangzhou, China; 3 Department of Maternal and Child Health, School of Public Health, Sun Yat-Sen University, Guangzhou, China

**Keywords:** Remote fetal heart rate self-monitoring, Wireless, Antenatal care, Obstetric outcomes, Cesarean section

## Abstract

**Background:**

Compared to traditional fetal heart rate monitoring (FHR) for the outpatients in clinic, remote FHR monitoring shows real-time assessment of fetal wellbeing at home. The clinical function of remote FHR monitoring in pregnant wome in outpatient is still unclear.

**Objective:**

To explore the feasibility of remote FHR self-monitoring in singleton pregnant women from southern China.

**Study design:**

This prospective cohort study was conducted at one tertiary center in southern China. Pregnant women used a mobile cardiotocogram device to measure the FHR at least once a week until delivery in the remote group. For the control group, pregnant women underwent traditional FHR monitoring once a week in the outpatient clinic. The rate of cesarean section, risk of postpartum hemorrhage and adverse neonatal outcomes were compared between the two groups. All the pregnant women completed a questionnaire survey to evaluate their acquisition of remote FHR self-monitoring.

**Results:**

Approximately 500 women were recruited in the remote FHR self-monitoring group (remote group), and 567 women were recruited in the traditional FHR monitoring group (control group). The women in the remote FHR monitoring group were more likely to be nulliparous (*P* < 0.001), more likely to have a higher education level (*P* < 0.001) and more likely to be at high risk (*P* = 0.003). There was no significant difference in the risk of cesarean section (*P* = 0.068) or postpartum hemorrhage (*P* = 0.836) between the two groups. No difference in fetal complications was observed across groups, with the exception of the incidence of NICU stays, which was higher in the remote group (12.0% vs. 8.3%, *P* = 0.044). The questionnaire survey showed that the interval time (*P* = 0.001) and cost (*P* = 0.010) of fetal heart rate monitoring were lower in the remote group. Regarding age, prepregnancy BMI, risk factors, education level, maternal risk and household income, senior high school (OR 2.86, 95% CI 1.67–4.90, *P* < 0.001), undergraduate (OR 2.96, 95% CI 1.73–5.06, *P* < 0.001), advanced maternal age (OR 1.42, 95% CI 1.07–1.89, *P* = 0.015) and high-risk pregnancy (OR 1.61, 95% CI 1.11–2.35, *P* = 0.013) were independent factors for pregnant women to choose remote fetal monitoring. Multiparty (OR 0.33, 95% CI 0.21–0.51, *P* < 0.001), full-time motherhood (OR 0.47, 95% CI 0.33–0.678, *P* < 0.001) and high household income (OR 0.67, 95% CI 0.50–0.88, *P* = 0.004) were negatively correlated with the choice of remote FHR self-monitoring.

**Conclusion:**

Remote FHR self-monitoring technology has a lower cost and shows potential clinical efficacy for the outpatient setting in southern China. This approach does not increase the risk of cesarean section or adverse neonatal outcomes. It is acceptable among nulliparous pregnant women with a high education level, high household income or high risk. Further research is needed to assess the impact of this technology on obstetric outcomes in different health settings.

## Introduction

Globally, more than 3.8 million perinatal deaths, including 2 million stillbirths, occur each year [1]. More than 98% of stillbirths occur in developing countries, which is 10 times greater than the rate in developed countries [1]. In southern China, the perinatal mortality was 13.5 per 1000 births in 2019 owing to the lack of continuous electronic fetal heart rate (FHR) monitoring [2]. FHR monitoring, which is the primary method for the early detection of abnormal fetal wellbeing, provides an opportunity for effective intervention to prevent neonatal morbidity and mortality [3]. In general, the traditional devices for FHR monitoring require pregnant women to go to the hospital, thus limiting their use to the clinic. This traditional FHR monitoring model also limits routine observations to pregnant women at one period and is restrictive to an examination couch. It is difficult for women who live far from the hospital to attend 1 or 2 times a week, as is recommended for women with a high-risk pregnancy [4]. Although the distances to the hospital are short in general, the travel time to reach the hospital is often unpredictable. In general, the time interval between the arrival of pregnant women at the care unit and the decision to perform surgery is more than 3 h, owing to the unpredictable travel time when pregnancies felt terrible. The above barrier to health care has been highlighted in the context of the COVID-19 pandemic [5].

Telemedicine, which is the exchange of information among physically distant clinical sites via telecommunications, enables health care across distances, particularly in rural areas, thereby avoiding unnecessary visits to tertiary centers [6]. Wireless remote FHR monitoring systems transmit fetal heart rate data to a central sever either through the web or Bluetooth, thus allowing real-time assessment of fetal wellbeing. Remote FHR monitoring have become a mainstream tendency. Pilot studies have demonstrated that this remote FHR monitoring system is feasible and acceptable among both pregnant women and obstetric outpatient clinics in developed countries [7]. To date, a wireless, remote fetal monitoring system composed of a wireless belt and acoustic sensor has been promoted as at-home fetal monitoring for high-risk pregnant women, especially in pregnant women who cannot move up to obstetric care for routine visits [8].

Remote fetal monitoring has become crucial in the management of high-risk pregnancies to improve the assessment of fetal well-being [9, 10]. The remote fetal monitoring system could reduce prenatal visits. A previous study demonstrated that a reduction in perinatal visits was recommended for low-risk pregnancies and did not increase adverse maternal or fetal outcomes [11]. Yvonne [12] reported that wireless remote fetal monitoring yields an equally satisfying experience as traditional perinatal care without an increase in perinatal complications. Recently, wireless remote fetal monitoring was recommended for pregnancies during the present COVID-19 pandemic. Rossignol [13] identified that electronic fetal monitoring increased the risk of cesarean section. However, compared to the traditional FHR monitoring, it’s controversial that increased use of remote fetal monitoring system, variance in interpretation and intervention led to increased rate of cesarean section.

Therefore, it is important to introduce a remote fetal monitoring method to empower pregnant women to perform fetal surveillance at home, thereby improving maternal and neonatal outcomes without increasing rate of cesarean section. Besides, the cost of antenatal care varies in different regions of China. Feng [14] reported that the frequency and contents of antenatal care was associated with regional differences, as well as the socio-economic status of the pregnant women in China. The purpose of the present study was to explore impact on delivery mode and cost effectiveness of the remote fetal monitoring system for antenatal care in rural areas of Guangdong Province.

## Material and methods

A prospective study evaluated the use of wireless remote FHR self-monitoring on obstetric outcomes compared to traditional FHR monitoring in a single tertiary center in southern China. The study was conducted in accordance with the Declaration of Helsinki. Ethical approval was obtained from the Ethics Committee of Sun Yat-sen University (No. 21-413) and Zhongshan Public welfare science and technology research Founadtion (No.2019B1012).

Singleton pregnancies without serious complications were eligible to participate in the study if they were 20 years of age or older, able to consent, and at 34 weeks of gestation or later. The exclusion criteria included multiple gestations, uncontrolled hypertension, preeclampsia, uncontrolled gestational diabetes mellitus and fetal abnormalities. Enrollment occurred from January 2021 through December 2021 at a tertiary referral maternity unit. Women were approached by trained research assistants in the antenatal setting. All women provided written informed consent to participate in this study. The demographic characteristics and medical history of all participants were reviewed by a doctor. High-risk was defined as the women complicated with advanced maternal age (≥ 35 years old), history of abnormal pregnancy, gestational hypertension, gestational diabetes mellitus, intrahepatic cholestasis of pregnancy, immune diseases such as systemic lupus erythematosus, rheumatoid arthritis, connective tissue disease, nephritis, heart disease, fetal growth restriction, placenta previa, beach position. If the participant underwent traditional FHR monitoring, FHR monitoring was applied for them once a week. The participant who took remote FHR monitoring, a minimal FHR monitoring frequency of one was required everyday. Once the abnormal results, including low variability, bradycardia, tachycardia, decelerations, and sinusoidal patterns, were identified, the patients were informed to visit a hospital.

The primary outcome was the rate of cesarean section. Secondary outcomes for pregnancies included gestational age at birth, postpartum hemorrhage (≥ 500 ml), and adverse neonatal outcomes. Adverse neonatal outcomes included fetal distress, neonatal asphyxia (Apgar score < 7 at 1 min), preterm birth (gestational age at delivery < 37 weeks), meconium-stained amniotic fluid, low birth weight (LBW < 2500 g), macrosomia (neonatal birth weight ≥ 4000 g), admission to the neonatal intensive care unit (NIUC) and neonatal death. In addition, a questionnaire survey about the satisfaction and cost of fetal monitoring, including the awareness of the importance of antenatal fetal heart rate monitoring, the interval time of taking non-stress test, the travel time and the travelling expense to hospital from home, the frequency of remote FHR monitrong, and the knowledge of antenatal fetal heart rate monitoring, was administered to patients to detect differences across groups. The study aimed to identify factors that affected the use of remote FHR self-monitoring.

### Statistical analysis

Categorical variables were summarized using frequency, presenting counts and relative percentages, and were compared by using Fisher’s exact test. Logistic analysis was used for the impact on willing of remote FHR monitoring, regarding education level, gradity, parity, career, high-risk status, pre-pregnancy BMI, rate of abortion, assisted reproduction. A 95% confidence interval (CI) was calculated for the willing rate. Statistical analysis was performed using SPSS software version 23.0 (Copyright, SPSS, USA).

## Results

**Fig. 1:**
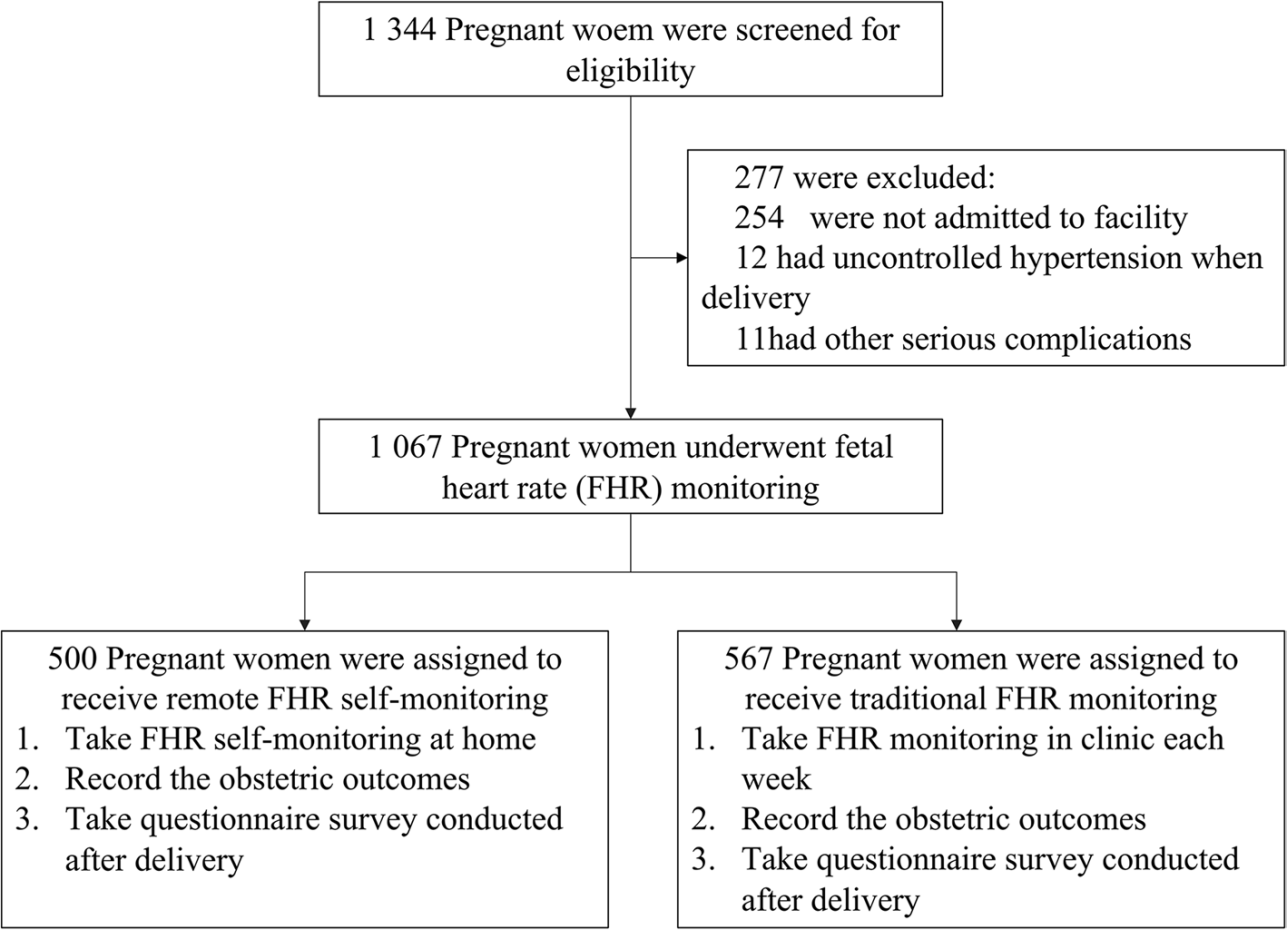
Enrollment and the flow of participants

From January 2021 through December 2021, a total of 1344 pregnant women were screened for eligibility. There were 254 cases admitted to outher facility, 12 cases suffered from uncontrolled hypertension at delivery, and 1 case complicated with serious preelampsia. A total 1067 pregnant women were enrolled in our study. None of them withdrew their consent or were lost to follow-up during the study period. There were 500 women in the remote FHR self-monitoring group (remote group) and 567 women in the traditional FHR monitoring group (control group). The flow of participants and completeness of data were similar across groups (Fig. 1).

Table 1 enumerates the clinical characteristics and social background between the two groups. There was a higher proportion of nulliparous women in the remote FHR group (58.0% vs. 44.6%, *P* < 0.001). Approximately 68.8% of the remote group had high-risk pregnancies, while 60.0% of the control group had high-risk pregnancies (*P* = 0.003). Compared to women in the control group, women in the remote group had a higher education level (*P* < 0.001). The proportion of full-time mothers was lower in the remote group (*P* < 0.001). The demographic characteristics, including age, prepregnancy BMI, gravidity, abortion, assisted reproduction and household income, were similar for these groups.

**Table 1:** The characteristics of pregnant women between remote group and control group

Features	Remote group (*N* = 500) (n, %)	Control group (*N* = 567) (n, %)	*Χ*^2^	*P*
Age (years old)			2.811	0.094
20 ~ 34	401(80.20)	477(84.13)		
≥ 35	99(19.80)	90(15.87)		
Pre-pregnancy BMI(kg/m^2^)			2.229	0.892
< 18.5	49(9.80)	53(9.35)		
18.5 ~ 23.9	363(72.60)	419(73.90)		
≥ 24.0	88(17.60)	95(16.75)		
Education level			19.155	< 0.001^**^
Junior school	22(4.40)	67(11.82)		
Senior school	193(38.60)	205(36.16)		
Undergraduate	285(57.00)	295(52.03)		
Parity			19.030	< 0.001^**^
Nulliparous	290(58.00)	253(44.62)		
Multiparous	210(42.00)	314(55.38)		
Abortion			1.389	0.239
0	317(63.40)	379(66.84)		
≥ 1	183(36.60)	188(33.16)		
Assisted reproduction			2.041	0.153
No	493(98.60)	552(97.35)		
Yes	7(1.40)	15(2.65)		
Career			12.426	< 0.001^**^
Working women	427(85.40)	436(76.90)		
Full-time mother	73(14.60)	131(23.10)		
Monthly income (Yuan)			0.445	0.505
< 5000	220(44.00)	238(41.98)		
≥ 5000	280(56.00)	329(58.02)		
High-risk			9.014	0.003^**^
No	156(31.20)	227(40.04)		
Yes	344(68.80)	340(59.96)		

High-risk includes advanced maternal age, history of abnormal pregnancy, obstetric complications (gestational hypertension, gestational diabetes mellitus, intrahepatic cholestasis of pregnancy, immune diseases such as systemic lupus erythematosus, rheumatoid arthritis, connective tissue disease, nephritis, heart disease, etc.), fetal growth restriction, placenta previa, beach position

*BMI* body mass index

**p<0.01

As shown in Table 2, the maternal and neonatal outcomes were summarized between the two groups. In the remote group, 37.6% of women underwent cesarean section, whereas 32.3% of women in the control group underwent cesarean section (*P* = 0.068). There was no significant difference in the incidence of postpartum hemorrhage (1.60% vs. 1.76%, *P* = 0.836). The incidence of admittion of NICU was higher in remote group compared to the control group (12.0% vs. 8.3%, *P* = 0.044). There was no significant difference in the risk of adverse neonatal outcomes, including fetal distress (4.60% vs. 3.35%, *P* = 0.295), neonatal asphyxia (4.005 vs. 1.235, *P* = 0.483), preterm birth (6.405 vs. 7.58%, *P* = 0.450), meconium-stained amniotic fluid (10.80% vs. 10.93%, *P* = 0.944), low birth weight (4.40% vs. 5.47%, *P* = 0.423), macrosomia (3.60 vs. 2.47%, *P* = 0.280) and neonatal death (0.20% vs. 0.71%, *P* = 0.379).

**Table 2:** The obstetric outcomes between remote group and control group

Features	Remote group (*N* = 500) (n, %)	Control group (*N* = 567) (n, %)	*Χ*^2^	*P*
Delivery mode			3.322	0.068
Vaginal birth	312(62.40)	384(67.72)		
Cesarean section	188(37.60)	183(32.28)		
Postpartum hemorrhage	8(1.60)	10(1.76)	0.043	0.836
Fetal outcomes				
Fetal distress	23(4.60)	19(3.35)	1.096	0.295
Neonatal asphyxia	4(0.80)	7(1.23)	0.492	0.483
Preterm birth	32(6.40)	43(7.58)	0.570	0.450
Low birth weight	22(4.40)	31(5.47)	0.641	0.423
Macrosomia	18(3.60)	14(2.47)	1.168	0.280
Fetal death	1(0.20)	4(0.71)	-	0.379
Addmission NICU	60(12.00)	47(8.29)	4.055	0.044^*^
Adverse neonatal outcomes	146(29.20)	150(26.46)	0.999	0.319

Adverse neonatal outcomes include fetal distress, neonatal asphyxia, preterm birth, low birth weight, mascrosomia, neonatal death and admission of NICU

^*^p<0.05

Table 3 shows the details of the questionnaire survey conducted after delivery. The interval time (*P* = 0.001) and cost (*P* = 0.010) of fetal heart rate monitoring in the clinic were lower in the remote group. Compared to women in the control group, pregnancies in the remote group have a more comprehensive knowledge of common sense regarding fetal heart rate monitoring, such as appropriate interval time, the optimal time and the frequency of fetal surveillance. There was no significant difference in awareness of the necessity of weekly fetal heart rate monitoring across groups.

**Table 3:** The result of questionnaire survey between remote group and control group

	Remote group (*N* = 500) (n, %)	Control group (*N* = 567) (n, %)	*Χ*^2^	*P*
Is it important to take fetal monitoring weekly?			1.368	0.505
No	19(3.80)	23(4.06)		
Important	271(54.20)	287(50.62)		
Most important	210(42.00)	257(45.33)		
How long to take non-stress test (NST)?			29.169	< 0.001^**^
< 30 min	425(85.69)	418(73.72)		
≥ 30 min	70(14.1)	131(23.10)		
No idea	1(0.20)	18(3.17)		
The travel time to go to the hospital for NST			10.648	0.001^**^
< 2 h	356(71.20)	350(61.73)		
≥ 2	144(28.80)	217(38.27)		
The travelling expense to go to the hospital for NST			6.682	0.010^*^
< 20 Yuan	244(48.80)	232(40.92)		
≥ 20 Yuan	256(51.20)	335(59.08)		
How often choose remote FHR monitoring?			266.407	< 0.001^**^
Doctor order	30(6.00)	155(27.34)		
Sometimes	125(25.00)	297(52.38)		
Often	345(69.00)	115(20.28)		
Do you need an empty stomach before FHR monitoring?			30.806	< 0.001^**^
No	487(97.40)	504(88.89)		
Yes	4(0.80)	36(6.35)		
No idea	9(1.80)	27(4.76)		

^*^p<0.05

^**^p<0.01

The influencing factors of choosing remote fetal heart monitoring were evaluated by logistic regression analysis (Table 4). Women with higher education levels, such as senior high school (OR 2.86, 95% CI 1.67–4.90, *P* < 0.001) and undergraduate (OR 2.96, 95% CI 1.73–5.06, *P* < 0.001), chose remote fetal heart rate monitoring. Moreover, advanced maternal age (OR 1.42, 95% CI 1.07–1.89, *P* = 0.015) and high-risk pregnancy (OR 1.61, 95% CI 1.11–2.35, *P* = 0.013) were independent factors for pregnant women to choose remote fetal monitoring. Multiparity (OR 0.33, 95% CI 0.21–0.51, *P* < 0.001), full-time mothers (OR 0.47, 95% CI 0.33–0.678, *P* < 0.001) and high household income (OR 0.67, 95% CI 0.50–0.88, *P* = 0.004) were negatively correlated with the choice of remote fetal heart rate monitoring. The prepregnancy BMI, abortion and assisted reproduction were not correlated with the choice of remote fetal heart rate monitoring.

**Table 4:** The Logistic analysis of impact on willing of remote FHR monitoring

Variable	*OR*	95% *CI*	*P*
Education level			
Junior school	1.00		
Senior school	2.86	(1.67,4.90)	< 0.001^**^
Undergraduate	2.96	(1.73,5.06)	< 0.001^**^
Parity			
Nulliparous	1.00		
Multiparous	0.33	(0.21,0.51)	< 0.001^**^
Career			
Working women	1.00		
Full-time mother	0.47	(0.33,0.67)	< 0.001^**^
High-risk			
No	1.00		
Yes	1.42	(1.07,1.89)	0.015^*^
Age (years old)			
20 ~ 34	1.00		
≥ 35	1.61	(1.11,2.35)	0.013^*^
Pre-pregnancy BMI (kg/m^2^)			
18.5 ~ 23.9	1.00		
≥ 24.0	1.05	(0.75,1.48)	0.771
< 18.5	1.00	(0.65,1.54)	0.995
Gradity			
1	1.00		
≥ 2	1.92	(1.14,3.25)	0.015^*^
Abortion			
0	1.00		
≥ 1	0.96	(0.67,1.38)	0.822
Assisted reproduction			
No	1.00		
Yes	0.45	(0.17,1.14)	0.093
Monthly income (Yuan)			
< 5000	1.00		
≥ 5000	0.67	(0.50,0.88)	0.004^**^

^*^p<0.05

^**^p<0.01

## Discussion

Our study revealed that after 34 gestational weeks, pregnant women were able to use a remote wireless CTG device, which is accurate and safe for self-monitoring of fetal heart rate (FHR) at home. The clinical efficacy of remote FHR self-monitoring is equivalent to that in the clinic without increasing the risk of cesarean section. Besides, remote FHR self-monitoring did not increase the risk of postpartum hemorrhage or adverse neonatal outcomes. In addition, the questionnaire survey that administered after delivery showed that most pregnant women who chose remote CTG devices spent less time and money on FHR monitoring and had a more comprehensive knowledge. Education level, household income, and party and occupational status were considered impact factors for the use of remote FHR self-monitoring in pregnant women.

Based on our findings, remote FHR self-monitoring could facilitate the improvement of antenatal care modes. During the COVID-19 pandemic, remote surveillance of fetuses should be robust and safe. The usability and accuracy of remote FHR self-monitoring at home, as well as its ability to improve maternal engagement and wellbeing, make it feasible to be used as a supplement to traditional antepartum care in the clinic. In accordance with the present study, Kerner and coworkers [15] investigated the readability of FHR tracings of 36 high-risk pregnancies with FHR self-monitoring and showed the feasibility of remote FHR self-monitoring at home. We also revealed that remote FHR monitoring did not increase the risk of cesarean section and postpartum hemorrhage in singleton gestations. Similarly, Tamaru [16] and coworkers reported the feasibility of using remote FHR monitoring. They analyzed 1278 records of remote FHR monitoring from 101 pregnant women at low risk and determined that the obstetric outcomes were not affected by the abnormal records of remote FHR monitoring, according to the JSOG system.

In addition, Monincx [17] and colleagues conducted a randomized controlled trial to assess fetal surveillance with remote FHR monitoring. A total of 76 pregnant women at high risk were enrolled in remote antenatal care, and 74 pregnancies were enrolled in hospital care. There was no difference in perinatal morbidity between these two groups. Our study also found that the risk of perinatal morbidity did not increase in pregnant women at high risk. Furthermore, the utility of remote FHR self-monitoring did not mitigate or increase adverse neonatal outcomes. Rather than proving perinatal benefits in light of improved fetal morbidity and mortality, our study revealed the feasibility of remote FHR self-monitoring. The usability results of the present study were consistent with those of a previously published study. A potential advantage of the use of remote FHR self-monitoring is that it is less likely to cause adverse maternal and neonatal outcomes. Remote FHR self-monitoring was found to support outpatient concerns, such as decreased fetal movement [8]. Therefore, the clinical benefits of remote FHR self-monitoring suggest that it should be expanded, and that additional rigorous research should be performed.

Not only the feasibility of remote FHR self-monitoring for pregnancy but also satisfaction and cost should be considered important factors in perinatal telemedicine. A retrospective cohort [18] of 77 pregnant women using remote FHR self-monitoring at home revealed that the usage and satisfaction were lower than those for traditional FHR monitoring in the clinic via a questionnaire survey. In contrast, Kerner [15] and colleagues administered a questionnaire survey regarding the anxiety status of pregnancies to determine the satisfaction of remote FHR monitoring at home and found that an absence of anxiety occurred in those pregnancies. Recently, facilities and expectations for the use of remote FHR self-monitoring were found in the majority of remote groups. Our survey also showed that the time and cost of FHR monitoring were lower in the remote group. In line with our findings, Fanelli [19] and coworkers revealed that FHR self-monitoring at home reduced the cost of fetal monitoring. Considering the equivalent impact of fetal surveillance and low cost, remote FHR self-monitoring may be a solution for antepartum care during the COVID-19 pandemic. Remote FHR self-monitoring seemed to have a potential impact on improving antenatal care and expanding the monitoring capacity in settings where bulky wired or traditional equipment is unreliable.

Our findings identified that remote FHR self-monitoring was highly acceptable in nulliparous pregnant women. The hypothesis was that nulliparous pregnancies might lack experience and have a sense of “fear of the unknowns” emanating from the traditional fetal monitoring model.According to the study by Shakarami [20], nulliparous women was at higher risk of developing anxiety and stress compared to multiparous women. Hence, remote FHR monitoring, providing real-time fetal wellbeing, might reduce fear in nulliparous women. Remote FHR self-monitoring is a home-based telemedicine device that enhances the satisfaction of pregnant women. For outpatients, remote FHR self-monitoring led to more freedom and satisfaction, particularly for pregnant women at high risk. This remote system allowed pregnant women at high risk to monitor fetal wellbeing at home without frequent ambulatory visits or hospitalization. Previous clinical trials [21] have demonstrated that remote FHR monitoring successfully produced output data in 90% of instances for pregnant women at home after labor induction. Compared to multiparous pregnancies, more nulliparous pregnancies preferred remote FHR self-monitoring. Unsurprisingly, education level and economic level were factors that impacted the usage of remote FHR self-monitoring. Pregnant women opting for the traditional FHR monitoring model were more likely to be multiparous and not receiving government assistance. These findings emphasize the need for economic growth prior to the popularization of remote FHR self-monitoring to more populations in different health settings. A cross-sectional observational study enrolled 55 pregnant women and 7 obstetrics to use remote FHR self-monitoring and identified that wireless FHR monitoring displayed a favorable role in a low-resource setting [22]. Furthermore, Ryu and coworkers [20] performed a remote maternal and fetal monitoring system for both high- and low-resource settings and demonstrated that this remote system was usable and safe. However, only wireless maternal monitoring, not fetal monitoring, was provided for the low-resource setting (*n* = 485) in this trial [20]. The implementation of remote FHR self-monitoring in low-resource settings is still challenging.

A strength of this study is the clinical benefit of remote FHR self-monitoring in preventing or mitigating adverse fetal outcomes from the outpatient setting of a large, urban tertiary health center, which has not been previously published. Similar to the traditional FHR monitoring mode in the clinic, we found that remote FHR self-monitoring was similar and did not increase the risk of adverse neonatal outcomes, regardless of high-risk or low-risk pregnant women. Notably, there is no established strategy to prevent fetal distress. At present, we have not proven that remote FHR self-monitoring is helpful in preventing fetal distress. Further larger multicenter randomized trials are needed to clarify whether remote FHR monitoring can improve neonatal outcomes and reduce infant health care costs. In addition, we showed that the rate of cesarean section did not increase in pregnancies with remote FHR self-monitoring, which is encouraging feedback, as it could be complementary to traditional antenatal care. In addition, we assessed the potential factors affecting the use of remote FHR self-monitoring among pregnant women, which has not been a focus in previous research. We revealed that parity, education level, household income and health risk were associated with the usage of remote FHR self-monitoring.

The present study has several limitations, including the population being recruited from a single tertiary center, with a higher level of familiarity with smartphone technology in urban cities. Conyer [21] and colleagues reported that wireless remote fetal telemonitoring increased the adherence to antenatal care in rural areas. Further study will be needed to investigate whether the clinical impact of remote FHR self-monitoring would be similar in more rural populations. In addition, we did not assess the acceptability and satisfaction of pregnant women, which has been widely studied before [23, 24].

## Conclusion

In summary, we highlight that remote fetal heart rate monitoring is a feasible, safe and effective assessment for antenatal care that does not increase the risk of cesarean section and adverse neonatal outcomes. Thus, remote FHR self-monitoring might overcome the limitations of traditional fetal monitoring models. When we expand the use of this technology across China, the education level and economic level of pregnant women should be considered. Additional larger, multicenter studies assessing the impact of remote FHR self-monitoring on obstetric outcomes in different health settings are needed.

## Data Availability

The datasets generated and/or analysed during the current study are not publicly available due patient privacy but are available from the corresponding author on reasonable request.
